# The effects of temporal neck cooling on cognitive function during strenuous exercise in a hot environment: a pilot study

**DOI:** 10.1186/s13104-015-1210-0

**Published:** 2015-05-30

**Authors:** Soichi Ando, Takaaki Komiyama, Mizuki Sudo, Akira Kiyonaga, Hiroaki Tanaka, Yasuki Higaki

**Affiliations:** Faculty of Sports and Health Science, Fukuoka University, Fukuoka, Japan; Graduate School of Informatics and Engineering, The University of Electro-Communications, 1-5-1 Chofugaoka, Chofu, Tokyo 182-8585 Japan; Graduate School of Sports and Health Science, Fukuoka University, Fukuoka, Japan; Fukuoka University Institute for Physical Activity, Fukuoka University, Fukuoka, Japan; Physical Fitness Research Institute, Meiji Yasuda Life Foundation of Health and Welfare, Tokyo, Japan

**Keywords:** Cognition, Exercise, Brain, Hot environment, Body temperature

## Abstract

**Background:**

Heat stress potentially has detrimental effects on brain function. Hence, cognitive function may be impaired during physical activity in a hot environment. Skin cooling is often applied in a hot environment to counteract heat stress. However, it is unclear to what extent neck cooling is effective for cognitive impairment during exercise in a hot environment. The purpose of this study was to examine the effects of temporal neck cooling on cognitive function during strenuous exercise in a hot environment.

**Methods:**

Eight male young participants (mean ± SD, age = 26.1 ± 3.2 years; peak oxygen uptake = 45.6 ± 5.2 ml/kg/min) performed Spatial delayed response (DR) task (working memory) and Go/No-Go task (executive function) at rest and during exercise in the Hot and Hot + Cooling conditions. After the participants completed the cognitive tasks at rest, they cycled the ergometer until their heart rate (HR) reached 160 beats/min. Then, they cycled for 10 min while keeping their HR at 160 beats/min. The cognitive tasks were performed 3 min after their HR reached 160 beats/min. The air temperature was maintained at 35°C and the relative humidity was controlled at 70%. Neck cooling was applied to the backside of the neck by a wet towel and fanning. We used accuracy of the Spatial DR and Go/No-Go tasks and reaction time in the Go/No-Go task to assess cognitive function.

**Results:**

Neck cooling temporarily decreased the skin temperature during exercise. The accuracy of the cognitive tasks was lower during exercise than that at rest in the Hot and Hot + Cooling condition (p < 0.05). There were no differences in the accuracy between the Hot and Hot + Cooling conditions (p = 0.98). Neither exercise (p = 0.40) nor cooling (p = 0.86) affected reaction time. These results indicate that temporal neck cooling did not alter cognitive function during strenuous exercise in a hot environment.

**Conclusions:**

The present study suggests that temporal neck cooling with a wet towel and fanning is not effective for attenuating impairment of working memory and executive function during strenuous exercise with a short duration in a warm and humid environment.

## Background

Many sports and occupational settings require high level cognitive ability under physiological stress in a hot environment. For example, soccer players make decisions quickly and appropriately while running in a hot environment. Occupational workers are often forced to carry out complex physical works in a hot summer. Physical activity in a hot environment imposes a severe burden on the cardiovascular system because of competition between the muscle, skin, and brain for the available systemic blood flow [1, 2]. It has been suggested that exercise in a hot environment may decrease arousal level [3], and the brain seems to be particularly vulnerable to hyperthermia [4]. Although our knowledge of cognitive function in a hot environment is mainly based upon data from a resting condition [5–9], it is likely that exercise in a hot environment impair cognitive function.

Skin cooling is known to reduce thermal strain and discomfort at rest and during exercise [5, 9–13]. Hence, skin cooling is often applied in a hot environment to counteract heat stress. Recent studies indicated that head cooling has the beneficial effects on short-term memory in a hot environment [6, 14]. Furthermore, neck cooling may enhance cognitive performance in complex tasks following exercise-induced hyperthermia [15]. However, it is unclear to what extent skin cooling has beneficial effects on cognitive function during exercise in a hot environment. In many sports and occupational settings, cognitive function plays an important role under physiological stress in a hot environment. Thus, it is imperative to examine the effects of skin cooling on cognitive function during exercise under various experimental conditions. In the present study, we used temporal neck cooling because brief neck cooling is easily available and practically applicable to situations where exercise or physical activity is actually performed in a hot environment. Furthermore, this maneuver does not interfere with performing the cognitive task. In the present study, we hypothesized that if brief neck cooling has beneficial effects on cognitive function during exercise in a hot environment, the impairment in cognitive function may be attenuated by physiological and/or psychological changes induced by brief neck cooling.

When exercise duration is prolonged in a hot environment, dehydration is inevitable. Dehydration may also impair cognitive function although the results in previous studies are somewhat controversial [16–24]. Thus, we used strenuous exercise with a short duration to exclude the possibility that dehydration also influences cognitive function. This allows us to focus on the effects of temporal neck cooling on cognitive function during exercise in a hot environment.

The purpose of the present study was to examine the effects of temporal neck cooling on cognitive function during strenuous exercise in a hot environment. This study will provide empirical evidence to demonstrate the effects of temporal neck cooling on cognitive function during strenuous exercise with a short duration in a hot environment, especially its validity and limitations.

## Methods

### Participants

Eight male young participants (mean ± SD, age = 26.1 ± 3.2 years; height = 1.75 ± 0.06 m; weight = 69.6 ± 8.3 kg; peak oxygen uptake = 45.6 ± 5.2 ml/kg/min) were fully informed of the risks and discomforts associated with this study before giving written informed consent to participate. The participants were free of cardiovascular, cerebrovascular, or respiratory disease and were not taking any medications. They were not acclimated to exercising in a hot environment. This study was approved by the ethics committee of Fukuoka University and was in accordance with the Declaration of Helsinki.

### Cognitive task

**Figure 1:**
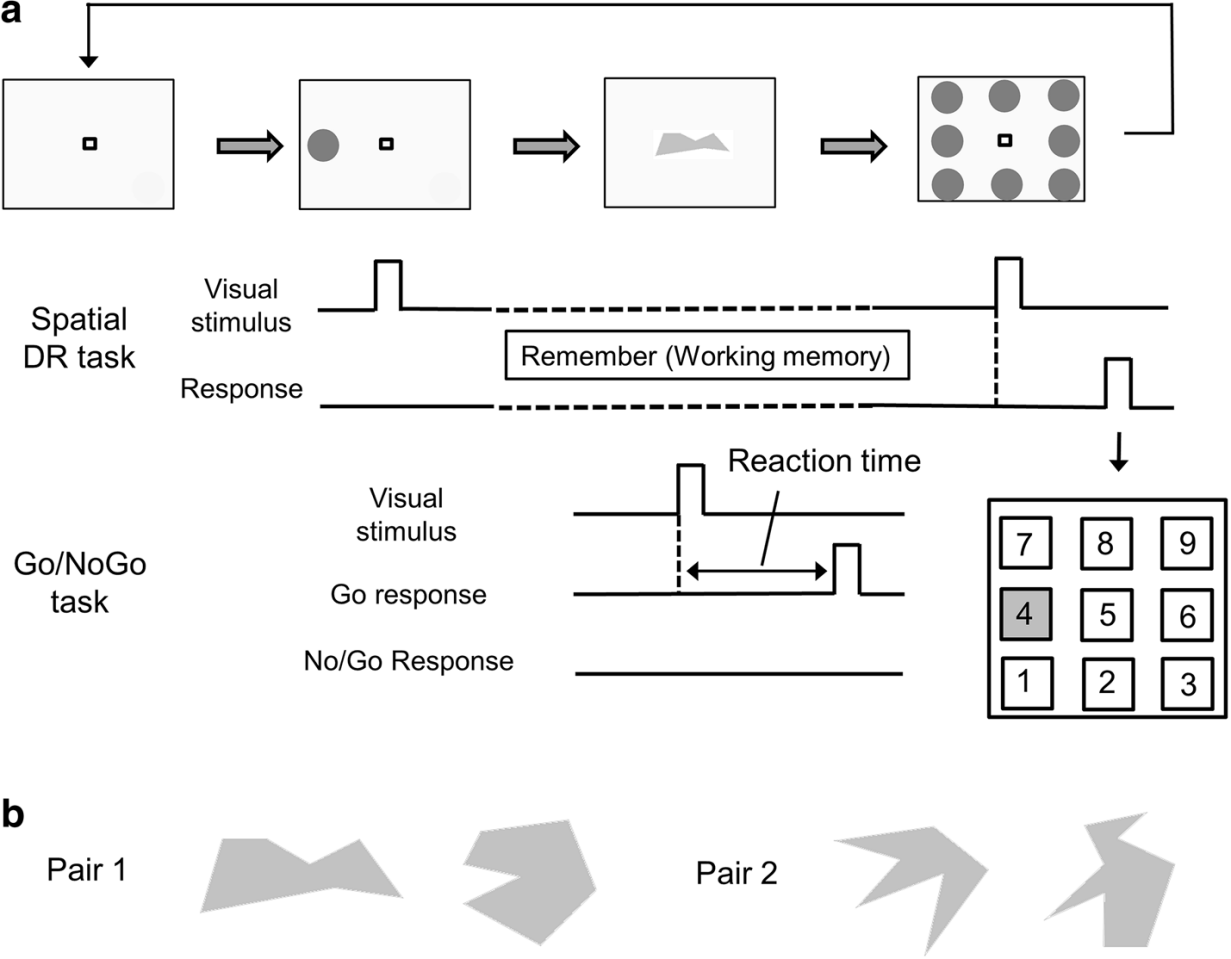
**a** Spatial delayed response (Spatial DR) task and Go/No-Go task. At the beginning of the Spatial DR task, a visual cue was presented at one of the eight locations. The participants remembered the location during the Go/No-Go task. After the Go/No-Go task, participants responded by pressing the button of the ten-key corresponding to the remembered location. In this case, participants had to press the number *4*. **b** Examples of the paired figures. One of the paired figures was randomly presented in the Go/No-Go task.

A laptop computer was used to present visual stimuli. The participants performed a combination of the Spatial delayed response (Spatial DR) task and the Go/No-Go task [25, 26], where working memory and executive function were required. The participants performed the cognitive task while they faced a computer display at a viewing distance of approximately 80 cm on a cycle ergometer (75XLII, COMBI Wellness, Tokyo, Japan). Details in the present cognitive task were described elsewhere [26], and were summarized in Figure 1a. In brief, at the beginning of the cognitive task, the participants remembered the location where visual stimulus was presented (Spatial DR task). Then, one of the paired figures (Figure 1b) was presented (Go/No-Go task). In the case of a Go-trial, participants released the shift button as quickly as possible. In the case of No-Go trials, participants continued pressing the shift button. After the Go/No-Go task, visual stimuli were presented at eight locations surrounding the fixation point. The participants pressed the button of the ten-key corresponding to the remembered location (Spatial DR task). The cognitive task continued until participants completed 30 trials of both tasks. The participants pressed the ten-key with the right index finger (see right bottom of Figure 1a) and pressed the shift button on the keyboard with the left index finger. The ten-key and keyboard were horizontally situated above both sides of the handlebar of the ergometer.

We used accuracy of the cognitive task and reaction time in the Go-trial to assess cognitive function. In the Spatial DR task, error trials were defined as incorrect responses to the remembered location. In the Go/No-Go task, error trials were defined as omissions of the response in the Go-trial or incorrect response in the No-Go trial. Response accuracy was calculated as the number of correct trials divided by the total number of trials. When the participants finished five successive trials in the Go/No-Go task, the relation between correct response and the figure was reversed from the next trial [27]. After the next five successive trials were completed, new paired figures were presented. Participants were unaware when a correct response and figure was reversed or when the new paired figures were presented in advance. Thus, we excluded trials immediately after the relation between the correct response and figure was reversed or one of the new paired figures was presented in the Go/No-Go task.

### Experimental procedure

The experiment was performed on two non-consecutive days. A few days before the first experiment, participants completed a series of practice blocks of the cognitive tasks at rest and during cycling until they were familiar with the task. The familiarization period continued until reaction times fall within 3 SD from the mean. All experiments were conducted inside an environmental control chamber (FHC-20S, Fuji-ika Sangyo, Chiba, Japan). In the Hot condition, the air temperature was maintained at 35°C and the relative humidity was controlled at 70%. In the Hot + Cooling condition, the air temperature and relative humidity were the same as the Hot condition, but neck cooling was applied to the backside of the neck by a towel wet in 21°C water and also by fanning from the back of the neck with a small electronic fan. Once the neck cooling was applied to the participant, the towel was put on until the experiment was completed. The towel was not changed during the experiment. The order of experimental conditions was assigned in a counterbalanced manner.

At the beginning of the experiment, the participants performed the cognitive task at rest. One minute after they completed the first cognitive task, participants started to cycle the ergometer. For the first 5 min, exercise intensity increased at 30–32 W/min in a ramp manner, and then increased at 20–21 W/min in a step manner until participants’ heart rate (HR) reached 160 beats/min. An ear sensor (COMBI Wellness, Tokyo, Japan), which was connected to the ergometer, continuously measured HR. Once the participants’ HR reached 160 beats/min, exercise intensity was adjusted automatically to maintain the target HR through the ear sensor. The participants cycled for 10 min while keeping their HR at 160 beats/min. We recorded exercise intensity and HR each minute during exercise after the participants’ HR reached 160 beats/min. The pedalling rate during cycling was chosen freely by each participant. Participants performed the cognitive task 3 min after their HR reached 160 beats/min in the Hot and Hot + Cooling conditions. In the Hot + Cooling condition, immediately after the recording of exercise intensity, HR, and skin temperature at 2 min after the participants’ HR reached 160 beats/min, neck cooling was started (approximately 2 min 10 s after the participants’ HR reached 160 beats/min). The participants did not drink beverages throughout the experiment.

### Other measurements

Skin temperature (O215-WT, Dretec, Saitama, Japan) was measured from the neck to evaluate the effects of the neck cooling. Probe of the thermometer was directly contacted to the backside of the neck. Skin temperature was recorded each minute during exercise after the participants’ HR reached the target level. Ratings of perceived exertion (RPE; 6–20 Borg scale) [28] were recorded immediately after each cognitive task (within 10 s). Blood lactate concentrations were measured at rest and immediately after exercise. Capillary blood was collected from the right earlobe. Blood lactate concentration was determined with the lactate oxidase method using an automated analyzer (Lactate Pro, Arkray, Kyoto, Japan). Body weight was measured without clothes before exercise after emptying the bladder, and after exercise. Weight reduction was expressed as a percentage of body weight, and dehydration was assessed by weight reduction. Body temperature was measured from the tympanic membrane before and after exercise. We measured body temperature at the room outside the environmental chamber. In the room outside the chamber, the air temperature was maintained at 21°C and the relative humidity was controlled at 50%.

### Data and statistical analysis

Two-way analysis of variance (ANOVA) with Exercise (rest and exercise) and Condition (Hot and Hot + Cooling) as the within-participants factors was performed for RPE, blood lactate concentration, body weight, body temperature, accuracy of the cognitive task, and reaction time in the Go-trial. We also performed two-way ANOVA with Time Course and Condition as the factors for exercise intensity, HR and skin temperature. Paired t tests were conducted to compare differences where appropriate. All data are expressed as mean ± SD. The significance level was set at p < 0.05.

## Results and discussion

### Exercise intensity and HR

Table 1 shows exercise intensity and HR after the participants reached a HR of 160 beats/min. We observed a significant main effect of Time Course (F [9,63] = 45.02, p < 0.001, η^2^ = 0.87) on exercise intensity. There were no significant main effect of Condition (F [1,7] = 1.62, p = 0.24, η^2^ = 0.19) and interaction (F [9,63] = 1.01, p = 0.44, η^2^ = 0.13). These results indicate that exercise intensity decreased as time elapsed and the degree of decrease was not different between conditions. Similarly, we found a significant main effect of Time Course (F [9,63] = 6.39, p < 0.001, η^2^ = 0.48) on HR. There were no significant main effect of Condition (F [1,7] = 2.91, p = 0.13, η^2^ = 0.29) and interaction (F [9,63] = 0.81, p = 0.61, η^2^ = 0.10). The significant main effect of Time Course may be ascribed to a slight higher HR around the first 3 min after the participants’ HR reached 160 beats/min. Overall, HR appears to be well controlled around the target level in the present study.

**Table 1:** Changes in intensity and HR during exercise after the participants’ HR reached 160 beats/min

	Time after the participants’ HR reached 160 beats/min (min)									
	1	2	3	4	5	6	7	8	9	10
Exercise intensity (W)										
Hot	164.5 ± 25.3	149.3 + 20.0	137.3 ± 29.9	132.5 ± 30.1	134.1 ± 27.7	130.4 ± 26.0	127.8 ± 27.4	127.9 ± 25.6	125.0 ± 29.8	123.9 ± 30.2
Hot + Cooling	163.3 ± 22.8	149.8 ± 29.8	136.6 ± 29.9	126.0 ± 34.0	125.6 ± 33.4	123.8 ± 33.8	121.9 ± 35.1	121.0 ± 36.1	117.1 ± 35.9	114.6 ± 35.6
Heart rate (beats/min)										
Hot	162.3 ± 1.7	163.5 ± 2.2	163.6 ± 2.3	161.3 ± 2.0	159.9 ± 1.3	161.4 ± 1.7	162.1 ± 2.1	160.3 ± 3.1	160.0 ± 1.3	160.3 ± 1.9
Hot + Cooling	162.6 ± 1.2	164.0 ± 3.0	165.1 ± 3.0	162.8 ± 1.7	160.8 ± 1.9	160.4 ± 1.6	161.1 ± 1.8	160.9 ± 1.4	161.0 ± 1.7	160.9 ± 1.3

Values are mean ± SD.

### RPE, blood lactate concentration, body weight, and body temperature

Table 2 shows RPE, blood lactate concentration, body weight, and body temperature. We found significant main effects of Exercise on RPE (F [1,7] = 174.49, p < 0.001, η^2^ = 0.96), blood lactate concentration (F [1,7] = 43.02, p < 0.001, η^2^ = 0.86), body weight (F [1,7] = 55.30, p < 0.001, η^2^ = 0.89), and body temperature (F [1,7] = 56.82, p < 0.001, η^2^ = 0.89). In contrast, Condition did not affect these parameters. There were no significant interactions. Collectively, exercise increased RPE and blood lactate concentrations to the same extent in both conditions. The degree of dehydration and increase in body temperature were not different between conditions. Indeed, we observed no difference in weight reduction between conditions (0.41 ± 0.07% in the Hot condition and 0.37 ± 0.22% in the Hot + Cooling condition).

**Table 2:** RPE, blood lactate concentration, body weight, and body temperature

Condition	Variable	Rest	Exercise	After
Hot	RPE	7.3 ± 0.4	15.9 ± 2.0*	
	Blood lactate concentration, mmol/l	1.0 ± 0.2		5.3 ± 1.9*
	Body weight, kg	69.1 ± 7.5	68.8 ± 7.5*	
	Body temperature, °C	36.1 ± 0.6	38.4 ± 0.4*	
Hot + Cooling	RPE	7.1 ± 0.9	15.8 ± 1.7*	
	Blood lactate concentration, mmol/l	1.0 ± 0.2		5.5 ± 1.7*
	Body weight, kg	68.9 ± 7.7	68.6 ± 7.7*	
	Body temperature, °C	36.5 ± 0.6	38.3 ± 0.3*	

Values are mean ± SD.

*RPE* ratings of perceived exertion.

* p < 0.001, vs. rest.

### Effects of neck cooling on skin temperature

**Figure 2:**
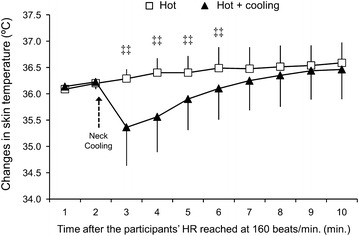
Neck skin temperature in the Hot and Hot + Cooling conditions during exercise. The *upper dashed arrow* indicates the time when neck cooling was started. ^‡‡^ *p* < 0.01 between the conditions.

Figure 2 illustrates the skin temperature during exercise. The main effects of Time Course (F [1,9] = 9.60, p < 0.001, η^2^ = 0.58) and Condition (F [1,7] = 8.03, p < 0.05, η^2^ = 0.53) were significant. There was a significant interaction between Condition and Time Course (F [9,63] = 8.88, p < 0.001, η^2^ = 0.56). These results indicate that skin temperature changed in a different manner between conditions. We observed significant differences in skin temperature between conditions after neck cooling. However, the effects of neck cooling were temporal and did not last until the end of the exercise.

### Cognitive function

**Figure 3:**
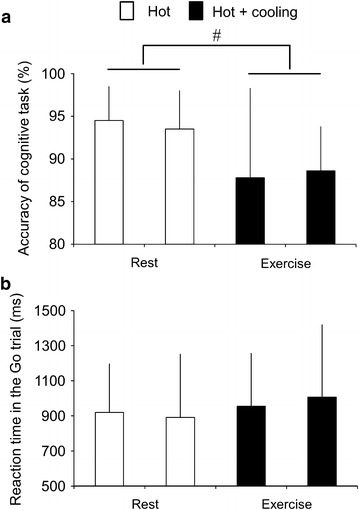
**a** Accuracy of the cognitive task at rest and during exercise. ^#^ *p* < 0.05 vs. rest. **b** Reaction time in the Go-trial at rest and during exercise.

Figure 3 illustrates the accuracy of the cognitive task (A) and reaction time in the Go-trial (B). We observed a significant main effect of Exercise on the accuracy of the cognitive task (F [1,7] = 8.78, p < 0.05, η^2^ = 0.56). We found neither a significant main effect of Condition (F [1,7] = 0.001, p = 0.98, η^2^ < 0.001) nor a significant interaction (F [1,7] = 0.27, p = 0.62, η^2^ = 0.04). These results indicate that the accuracy of the cognitive task significantly decreased during exercise, and that temporal neck cooling had no effects on the accuracy of the cognitive task. Reaction time in the Go-trial was not affected by Exercise (F [1,7] = 0.802, p = 0.40, η^2^ = 0.10) or Condition (F [1,7] = 0.03, p = 0.86, η^2^ = 0.005) in the present study.

To our knowledge, no studies have examined the effects of temporal neck cooling on cognitive impairment during strenuous exercise in a hot environment. We tested if temporal neck cooling with a wet towel and fanning has beneficial effects on cognitive function during strenuous exercise with a short duration in a hot environment. The main finding of this study was that temporal neck cooling did not attenuate the detrimental effects on cognitive function during strenuous exercise in a hot environment. The present finding suggests that temporal neck cooling with a wet towel and fanning is not effective enough to attenuate cognitive impairment during strenuous exercise with a short duration in a warm and humid environment.

We assessed cognitive function using the combination of spatial DR task and Go/No-Go task. The Spatial DR task requires working memory [25, 29]. The Go/No-Go task requires executive function, including selective attention, response inhibition and interference control [30, 31]. The prefrontal and parietal cortices play a key role in spatial working memory [32–35]. Activation in the prefrontal cortex is crucial for executive function [36, 37]. Furthermore, the frontopolar cortex, which is located at the most anterior part of the frontal cortex, is selectively activated in multi-tasking where execution of the primary task was maintained in a pending state while performing a secondary task [38]. Given that the present cognitive task was a modified version of multi-tasking, the present cognitive task required activation of the frontopolar cortex. Taken together, we can assume that the present cognitive task was appropriate to assess higher cognitive function in humans.

In the present study, HR was maintained at 160 beats/min during exercise. Indeed, RPE was approximately 16 immediately after the cognitive tasks during exercise. Furthermore, blood lactate concentration increased above 5 mmol/l after exercise in both conditions. Hence, in the present study, we can assume that exercise intensity was classified as strenuous.

Skin cooling may reduce thermal strain and/or discomfort at rest and during exercise [5, 9–13]. Thus, physiological as well as psychological changes induced by skin cooling may somehow attenuate the impairment in cognitive function during exercise in a hot environment. Previous studies indicated that head and neck cooling with frozen packs prevented the impairment of short-term memory [14, 39]. In the present study, the accuracy of the cognitive task decreased during exercise in the Hot and Hot + Cooling conditions. We observed no differences in the accuracy of the cognitive task during exercise between conditions. These results suggest that temporal neck cooling for a short period did not have beneficial effects on cognitive function during exercise in a hot environment under the present condition. In the present study, while neck cooling temporarily decreased skin temperature, neck cooling did not alter body temperature. These results mean that the neck cooling applied in this study was not effective enough to decrease body temperature. The present results are in line with the findings showing that skin cooling did not reduce body temperature [9–11, 40]. Moreover, we found no differences in RPE between conditions. Therefore, the lack of beneficial effects of temporal neck cooling on cognitive function would be ascribed to that physiological and/or psychological changes induced by brief neck cooling was not sufficient enough to attenuate the detrimental effects on cognitive function.

Dehydration is one of the potential factors that may impair cognitive function in a hot environment. Hence, we used strenuous exercise with a short duration. We expected that severe dehydration is unlikely to occur even in a hot environment in the present study. Indeed, weight reduction was less than 0.5% in the Hot and Hot + Cooling conditions. Previous studies suggested that dehydration may impair complex cognitive tasks when weight reduction was greater than 2% [21, 22, 39]. Indeed, Gaoua et al. [39] suggested that, when weight reduction did not exceed 0.5%, fluid replacement is adequate and dehydration does not alter cognitive function. Hence, we can assume that the effects of dehydration on cognitive function were negligible in the present study.

In contrast to the accuracy of the cognitive task, reaction time in the Go-trial was not altered by exercise. These results suggest that the effects of strenuous exercise in a hot environment are different between accuracy and speed of response. Previous studies indicated that nerve conduction velocity increases in the hot environment [14, 39]. Thus, exercise in a hot environment may increase in nerve conduction velocity, which leads to the decrease in reaction time. However, in the present study, it is unclear whether reaction time was not affected by strenuous exercise or whether increases in conduction velocity in a hot environment was not effective, possibly due to a short duration of exercise. Furthermore, we cannot rule out the possibility that increases in nerve conduction velocity compensated for the negative effects of strenuous exercise on speed of response. Provided that multiple factors are responsible for changes in response speed during exercise, further studies will be required to elucidate these points.

It has been suggested that increase in arousal level induced by exercise affect cognitive function [41, 42]. From this viewpoint, the present results may suggest that arousal level went beyond the optimal level during strenuous exercise in a hot environment, and that temporal neck cooling was not effective to alter arousal level. However, physiological mechanisms underlying the alterations in arousal level during exercise remains to be elucidated. Thus, further studies are required to understand the association between arousal level and cognitive function.

## Limitation

Whereas the present study benefited from a simple experimental design, there are several limitations in the present study. First, we matched exercise intensity during exercise based on the participants’ HR. Thus, the present results may be ascribed to the HR matched exercise. This point is a limitation to extend this finding to exercise-cognition interaction in other experimental conditions. Second, we used neck cooling by a wet towel and fanning because this method is practical and easily applicable. Once the wet towel was applied to the backside of the neck, the wet towel was not changed. Thus, the decrease in skin temperature was small and did not last more than 5 min (Figure 2). In contrast, in the previous studies that examined the effects of skin cooling on cognitive function, three cool frozen packs with a protective layer were applied to the head and neck [14, 39]. In another study, neck cooling was applied with neck cooling collar, and the cooling component was drained and replaced with a gel refrigerant [15]. Hence, cooling method in the present study was far less effective to counteract heat stress as compared with the method in the previous studies [14, 15, 39]. Furthermore, a previous study suggested that forehead cooling leads to the most beneficial effects on reducing thermoregulatory responses to a hot environment [43]. It is also suggested that the beneficial effects of head cooling may be more efficient with cognitive functions primarily involving the frontal cortex [39]. Accordingly, it is plausible that the absence of beneficial effects of neck cooling on cognitive function is attributable to the cooling method and cooled location in the present study. Future studies are needed to elucidate how skin cooling alters cognitive function during exercise in a hot environment with more effective cooling methods, including forehead cooling. Third, in the present study, exercise duration was short to avoid dehydration. Further investigation is necessary to clarify the effects of exercise with a longer duration in a hot environment on cognitive function. Finally, we measured tympanic temperature to evaluate body temperature. However, it is well known that tympanic temperature is not accurate as compared with rectal or oesophageal temperatures. Thus, accurate measurements of body temperature would be needed to reveal how and why cognitive function is altered during exercise in a hot environment.

## Conclusion

We examined the effects of temporal neck cooling with a wet towel and fanning on cognitive function during strenuous exercise with a short duration in a hot environment. The present study suggests that temporal neck cooling by a wet towel and fanning may not be effective for attenuating impairment of working memory and executive function during strenuous exercise with a short duration in a hot and humid environment.
